# Anticoagulation and Stroke

**DOI:** 10.1590/0004-282X-ANP-2022-S132

**Published:** 2022-08-12

**Authors:** Gisela Tinone, Mauricio Hoshino, Leandro Lucato, Luiz Roberto Comerlatti

**Affiliations:** 1 Universidade de São Paulo, Faculdade de Medicina, Departamento de Neurologia, São Paulo, SP, Brazil.; 2 Universidade de São Paulo, Faculdade de Medicina, Departamento de Radiologia, São Paulo, SP, Brazil.

**Keywords:** Ischemic Stroke, Cerebral Infarction, Anticoagulants, Embolic Stroke, Atrial Fibrillation, Cerebral Hemorrhage, AVC Isquêmico, Infarto Cerebral, Anticoagulantes, AVC Embólico, Fibrilação Atrial, Hemorragia Cerebral

## Abstract

In 2019, the American Heart Association did not recommend the emergent use of anticoagulation to prevent recurrence or progression of acute ischemic stroke. However, its indication in patients with extracranial artery intraluminal thrombus with artery-to-artery cerebral embolization must be analyzed. In this article, we will also discuss other indications of anticoagulation. This treatment could be indicated in patients with ischemic stroke caused by embolization from cervical artery dissection, catastrophic antiphospholipid antibodies syndrome (APS) and some cases of Covid 19. For secondary prevention, anticoagulation is recommended for Cardioembolic stroke such as nonvalvular atrial fibrillation and other cardiopathies, some patients with cervical artery dissection, stroke associated with cancer, and thrombophilia such as APS. The timing to restart anticoagulation after a large ischemic stroke or after a cerebral hemorrhagic transformation always represent a challenge. Even in patients with high risk of thromboembolism it should be delayed at least two weeks, ideal after four weeks.

## Resumo

Anticoagulação na fase aguda do acidente vascular isquêmico (AVCI) ainda é um tema bastante controverso. Em 2019, a American Heart Association (AHA) não recomendou o uso precoce da anticoagulação para evitar a progressão ou recorrência de AVCIs de grandes artérias. Mas sugere que a anticoagulação em pacientes com AVCI por embolização a partir de trombos intraluminais aderidos a parede de vasos extracranianos fosse analisada. Tanto a antiagregação como anticoagulação são opções terapêuticas nos casos de AVCI por dissecção arterial cervical. Mas em pacientes com AVCI por mecanismo de embolização, a anticoagulação poderia ser indicada. Pacientes com AVCI e sindrome catastrófica por anticorpos antifosfolípides devem ser anticoagulados além de receber tratamento específico. Outra indicação seriam casos de trombofilia como Covid 19. Nesse artigo de revisão será discutida a prevenção secundária de AVCI em situações específicas (AVCI cardioembólico em pacientes com fibrilação atrial não valvular ou outras cardiopatias, AVCI em casos oncológicos, além de outras trombofilias), além do período ideal para se introduzir ou reiniciar a anticoagulação após transformação hemorrágica.

## INTRODUCTION

Stroke represents one of the most important causes of death in many countries and the second in Brazil. In 2019, 101.074 Brazilians died of a stroke^1^. 

The incidence of stroke in our country is around 105 to 137 cases per 100,000 individuals per annum^12^. 

Stroke is a major cause of disability. Around 15-30% of survivors have severe disabilities^3^.

Stroke represents a significant public health problem globally.

According to Benjamin et al., more than 795,000 individuals in the United States have a stroke every year; about 610,000 are first or new strokes. Nearly one in four patients have had a previous stroke^4^.

Acute ischemic stroke treatment is crucial to minimize the burden of stroke, while secondary prevention therapy is also needed to avoid stroke recurrence. 

The treatment of hyperacute ischemic stroke focuses on reperfusion therapy of ischemic cerebral tissue with thrombolytic drugs and, more recently, mechanical thrombectomy.

Unfortunately, stroke registries have found that only 15% to 32% of patients presenting with ischemic stroke arrive at the hospital within three hours of symptom onset and only 40% to 50% of these are eligible for tPA (Alteplase). Even for mechanical thrombectomy within six hours, a single-center study estimated that only 1.7-2.5% of patients would be eligible for this treatment^5^. 

General supportive care with the management of physiological factors such as blood pressure, oxygenation, control of glycemia, avoiding dehydration and hyperthermia are essential in all patients^6^.

Anticoagulation therapy in acute ischemic stroke may be a therapeutic option in selected patients. This article will discuss some controversies on this topic but also emphasize the benefit of anticoagulation for secondary prevention in cardioembolic stroke and other situations.

## ANTICOAGULATION IN ACUTE ISCHEMIC STROKE

Emergent anticoagulants have been used in acute ischemic stroke, but there are controversies about their efficacy, the type of anticoagulant, route of administration, and treatment duration. 

In the TOAST trial, anticoagulation with intravenously low molecular weight heparinoid, ORG 10172 (danaparoid sodium) was evaluated in acute ischemic stroke patients. It was a randomized, double-blind, placebo-controlled, multicenter trial and enrolled 1281 individuals in the United States. The treatment included a seven-day course of danaparoid sodium or placebo with an initial bolus. Doses of heparinoids were adjusted according to anti-factor Xa activity. After seven days more patients treated with anticoagulation presented favorable outcomes but this benefit was not observed after three months. In the group receiving anticoagulation treatment, more patients presented intracranial bleeding complications. The authors' conclusions were that, despite the initial positive response to treatment at seven days, emergent treatment with anticoagulation was not associated with clinical improvement at three months^7^. 

In another study, the FISS-tris-trial patients with acute stroke with large artery occlusion were treated with anticoagulation with LMWH (fraxiparine) or aspirin. When analyzing subgroups of stroke, patients with symptomatic stenosis in the posterior circulation could benefit from treatment with LMWH anticoagulation^8^.

The IST trial compared patients with acute ischemic stroke treated with aspirin or anticoagulation with subcutaneous heparin. Treatment with aspirin produced a small reduction of deaths and recurrence of stroke without a significant increase in cerebral hemorrhage. Patients treated with subcutaneous heparin used a two-dose regimen: 12.500UI or 5.000UI twice daily. No benefit was observed in either subcutaneous heparin treatment. Fewer recurrent strokes were observed after 14 days but there was also an increase in hemorrhagic stroke and extracranial bleeding^9^.

A meta-analysis of IST, TOAST and FISS-tris describes a 0.8% risk of cerebral hemorrhage complications with the use of heparinoids without the benefit of anticoagulation in the stroke's acute phase^10^.

 American Heart Association guidelines do not recommend urgent anticoagulation in acute ischemic stroke in avoiding its recurrence or halting progression, since meta-analysis studies have shown a lack of benefit for anticoagulation treatment in these patients^6^.

 In patients with ischemic stroke associated with severe stenosis of an internal carotid artery, urgent anticoagulation is not recommended (AHA Guidelines 2019)^6^. However, some small studies have shown that for patients with extracranial artery intraluminal thrombus associated strokes, intravenous heparin or LMWH could be of benefit^11^ ^,^ ^12^.

We recommend anticoagulation with intravenous unfractionated heparin for some patients with embolization from carotid artery intraluminal thrombus or from basilar artery.

### Cerebral venous thrombosis

Patients with acute cerebral venous thrombosis are treated with anticoagulation but this topic will be discussed in another chapter.

### Cervical artery dissection

Cervical artery dissection is a common cause of stroke in young patients. Intramural hematoma can cause arterial stenosis or vessel occlusion. Cerebral infarction caused by cervical artery dissection may be a consequence of a hemodynamic mechanism or artery-to-artery embolization. Patients with moderate or severe arterial narrowing (intramural hematoma) associated with cerebral embolization could benefit from emergent anticoagulation with unfractionated heparin or LMW heparin. 

The CADISS (Cervical Artery Dissection in Stroke Study) group performed a randomized trial with 250 patients with extracranial carotid or vertebral artery dissection. When comparing anticoagulation and antiplatelet treatment there is no significant difference in outcome in Cervical Dissection patients^13^.

The presence of HITS (High-intensity transient signal) that represents embolic events occurrence in transcranial doppler monitoring may be another indication of anticoagulation in cervical artery dissection patients^13^. Large stroke cerebral infarcts could present hemorrhagic transformation therefore we avoid emergent anticoagulation in these situations. 

According to AHA guidelines (2019) patients with acute ischemic stroke and extracranial carotid or vertebral arterial dissection may be treated with antiplatelet or anticoagulation for a period of three to six months^6^.

### Thrombophilia and stroke

Patients with thrombotic states, such as in SARS-Cov-2 patients, antiphospholipid antibodies syndrome and other causes of thrombophilia could present thrombotic cerebral infarctions^14^ ^,^ ^15^. Except for patients with large strokes, anticoagulation could be started in the acute period. 

### Case 1

**Figure 1.**
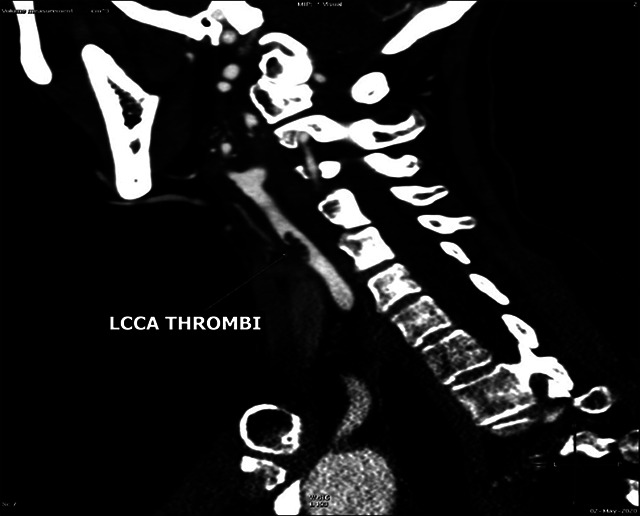
Sagittal reformatted CTA image shows the thrombus floating in the lumen of the left common carotid artery (arrow).

**Figure 2.**
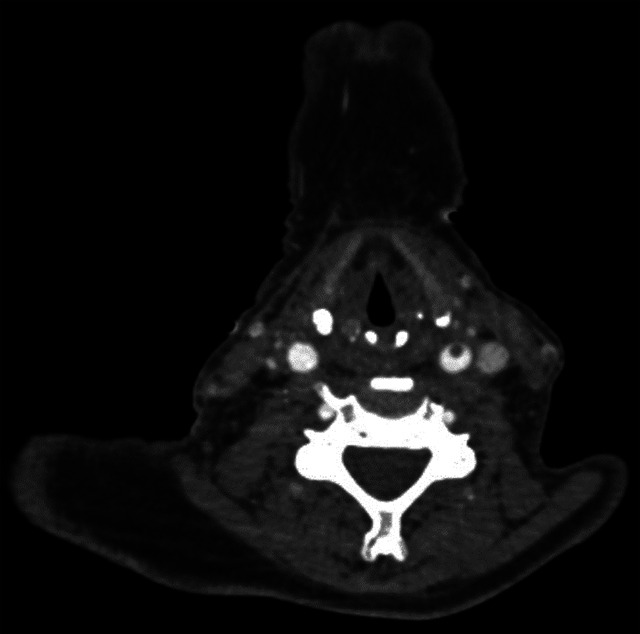
Axial reformatted CTA image also shows the luminal thrombus in the left common carotid artery.

**Figure 3.**
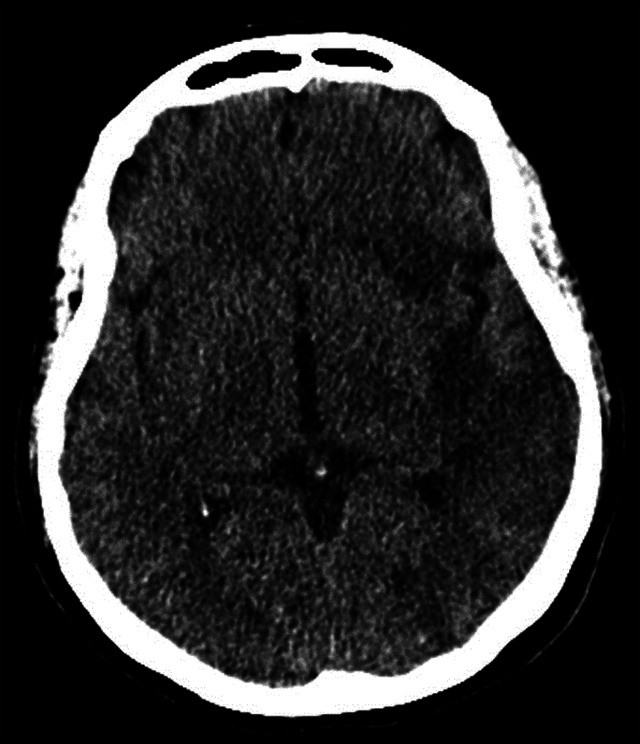
Follow-up head CT depicts the ischemic area in the left hemisphere more clearly as a hypoattenuation involving the left insula and frontal lobe.

**Figure 4.**
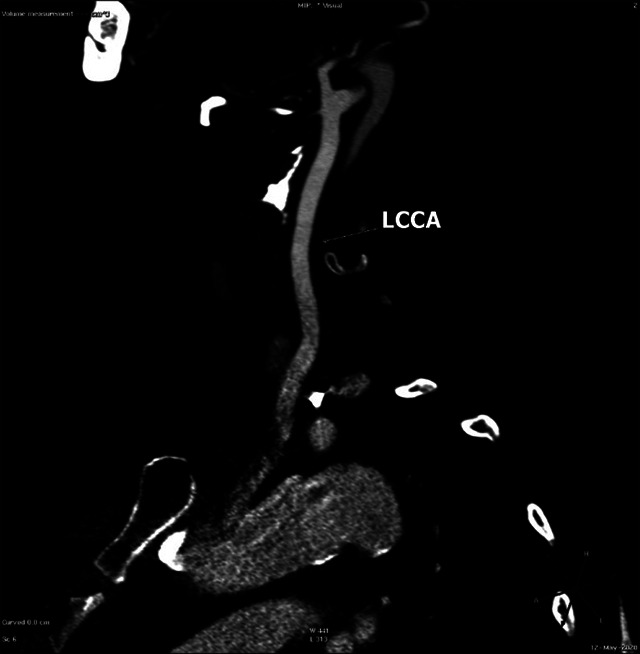
Follow-up sagittal reformatted CTA image shows the complete reabsorption of the thrombus.

In August 2020, a 60-year-old female, was admitted to our institution presenting flu-like symptoms (fever, cough, odynophagia) and dyspnea. She had a positive SARS-Cov-2 PCR test. Two days later, she suddenly presented subtle right hemiparesis and aphasia. Head Computer tomography (CT) showed no evidence of cerebral hemorrhage, and the patient was treated with intravenous thrombolysis (alteplase). CT-angiography evidenced a thrombus in the left common carotid artery (Figure 1 and 2). After 24 hours she had a further head CT (Figure 3) and intravenous unfractionated heparin was started. She received warfarin for three months. Follow-up CT-angiography (Figure 4) showed recanalization and anticoagulation was discontinued.

## SECONDARY STROKE PREVENTION TREATMENT

Around 20%of ischemic strokes are cardioembolic. Nonvalvular atrial fibrillation is one of the main causes of these strokes. Atrial fibrillation (AF) increases stroke risk three to five fold. Other cardioembolic mechanisms include: left atrial or ventricular thrombus, acute anterior ST-segment elevation myocardial infarction with** **anterior/apical akinesis or dyskinesis, mechanical left ventricular assist device, left ventricular ejection fraction less than 35%, and valvular heart disease including rheumatic mitral valve disease or mechanical prosthetic heart valves in the aortic or mitral position. In all of these cases, treatment with anticoagulants is recommended^16^.

### Anticoagulation in atrial fibrillation (AF) and stroke

AF predisposes the formation of thrombi-related left atrial appendage stasis and represents an important cause of cardiac embolism. Treatment with anticoagulation reduces the stroke risk associated with AF. A meta-analysis study including eight trials with 9598 patients with chronic AF, vitamin K antagonist (VKA) anticoagulants (warfarin) significantly reduced the risk of ischemic stroke (odds ratio (OR) 0.68, 95% confidence interval (CI) 0.54 to 0.85), by about one third, compared with aspirin (reduction of 60% with anticoagulation and 20% with aspirin) ^17^. 

However, anticoagulation increased the number of hemorrhagic complications. The International normalized ratio (INR) for VKA anticoagulation in patients with nonvalvular AF and prior ischemic stroke or transient ischemic attack (TIA) is recommended at a range between 2.0 and 3.0^16^.

More recent studies have evidenced the efficacy of Direct Oral Anticoagulants (DOAC): direct thrombin inhibitors (dabigatran) and inhibitors of factor Xa (rivaroxaban, apixaban, and edoxaban) anticoagulants. In trials with patients with AF, DOACs were compared with warfarin and they performed similar benefits, reducing ischemic stroke risk with a lower incidence of hemorrhagic cerebral complications. These studies compared DOAC and warfarin: ARISTOTLE (apixaban vs. warfarin in atrial fibrillation), RE-LY (dabigatran vs. warfarin in atrial fibrillation), ROCKET-AF (rivaroxaban vs. warfarin in atrial fibrillation), and ENGAGE AF-TIMI (edoxaban vs. warfarin in atrial fibrillation) ^18^ ^-^ ^21^.

The risk of strokes related to AF can be estimated using CHA_2_DS_2_-VASc Score^22^.

CHA_2_DS_2_VASc Score includes eight items (Table 1):

- congestive heart failure or left ventricular systolic dysfunction with ejection fraction <40% (one point);
- hypertension (one point);
- age ≥75 (doubled = two points);
- diabetes (one point);
- stroke (doubled = two points);
- vascular disease (one point);
- age 65 to 74 (one point) and;
- sex category (female = one point).

**Table 1.** CHA_2_DS_2_VASc score.

Condition	Points
Congestive heart failure	1
Hypertension	1
Age ≥ 75	2
Diabetes	1
prior Stroke or TIA	2
Vascular disease	1
Age 65-75	1
Sex	1
MAXIMUM SCORE	9

This score varies from 1 to 9. A patient with CHA_2_DS_2_-VASc Score 9 has an annual risk of 15.2% of presenting a stroke.

European Society of Cardiology guidelines for Atrial Fibrillation recommend stratifying patients with AF into three groups according to CHA_2_DS_2_-VASc score: score 0 - low risk, score 1 - medium risk, score ≥2 - high risk of presenting a stroke^23^.

In Table 2 we could observe that the stroke risk increased if a patient with AF presented other risk factors. According to European Society of Cardiology guidelines, patients with AF and CHA_2_DS_2_VASc Score ≥ 2 should be anticoagulated. The decision to initiate anticoagulation for a determined stroke patient must consider not only the stroke risk recurrence but also the risk of hemorrhagic complications^23^ ^-^ ^25^.

**Table 2.** CHA_2_DS_2_VASC score and stroke risk.

CHA_2_DS_2_VASC score	Stroke risk %/year
0	0
1	1.3
2	2.2
3	3.2
4	4.0
5	6,7
6	9.8
7	9.6
8	12.5
9	15.2

### Risks and prevention of bleeding with oral anticoagulants

There is no anticoagulant therapy without an increased risk of bleeding.

The studies showed that the pathogenesis of anticoagulant-associated bleeding included loss of vascular wall integrity and microbleeds related to microscopic pseudoaneurysm formation. When analyzing the risk of bleeding in anticoagulant therapy, the established risk factors are: type of anticoagulant, dose level, age, non-white race, prior bleeding, prior stroke, hypertension, cerebral amyloid angiopathy, liver disease, kidney disease, diabetes, oncologic diseases and disturbances of hemostasis^26^.

If someone considers these risk factors before choosing an anticoagulant therapy, the risk of serious bleeding could be reduced.

A number of bleeding risk scores have been validated, identifying potentially modifiable risk factors; however, the association is not causation and too many factors act as a cause and also a risk factor for anticoagulation.

The HAS-BLED (Table 3) score was derived from a Euro Heart Study, only with patients with atrial fibrillation and its use has been recommended in European and Canadian guidelines^26^.

**Table 3.** HAS-BLED score.

Letter	Clinical characteristic	Points
H	Hypertension (ie, uncontrolled blood pressure)	1
A	Abnormal renal and liver function (1 point each)	1 or 2
S	Stroke	1
B	Bleeding tendency or predisposition	1
L	Labile INRs (for patients taking warfarin)	1
E	Elderly (age greater than 65 years)	1
D	Drugs (concomitant aspirin or NSAIDs) or excess alcohol use (1 point each)	1 or 2
		Maximum 9 points

Other scores: ATRIA score, VTE-BLEED, HEMORR2HAGES score.

### When to initiate anticoagulation after a stroke

According to Hart et al., within two weeks of a stroke associated with nonvalvular atrial fibrillation (AF), the risk of developing an early recurrent cerebral embolism is calculated at around 0.1% and 1.3% per day^25^.

However, the time for introducing anticoagulation therapy remains unclear.

The RAF study evaluated 1029 patients in five groups: Low Molecular Weight Heparin (LMWH) alone, vitamin K antagonists (VKA) anticoagulants, direct oral anticoagulants, LMWH followed by VKA anticoagulants, and 449 were treated with antiplatelets^27^.

The study concluded that the recurrence of ischemic events (ischemic stroke or TIA) was 7.6% and symptomatic cerebral bleeding was 3.6% with 1.4% of major bleeding.

In patients with CHA_2_DS_2_-VASc score 4 to 8, the annual risk of stroke recurrence was higher and varied between 9.88% and 20.3%.

When analyzing the composite clinical events (risk of ischemic stroke recurrence and major bleeding), high CHA_2_DS_2_-VASc score, high NIHSS (National Institute of Health Stroke Scale), large lesion size, and type of anticoagulant were predictive factors^27^.

 RAF study recommends initiating anticoagulant therapy to avoid ischemic stroke recurrence between four and 14 days, except for patients with large ischemic lesion size associated with cerebral hemorrhage (hemorrhagic transformation - HT). In these patients, anticoagulation should be delayed. When anticoagulation was started within 30 days in patients with large lesion sizes, it was associated with a higher risk of complications. This is the main risk factor for HT^27^.

Even in moderate size ischemic stroke lesions, cardioembolic stroke is prone to present HT. Hornig et al. in a Magnetic Resonance Image study (MRI) showed that HT was present in 68.6% (24) of the 35 patients with Cardioembolic stroke. The risk of HT was associated with ischemic stroke volume (≥ 10ml). Hemorrhages are common in medium-size and large cardioembolic infarcts^28^.

Mac Grory et al. suggest the resumption of oral anticoagulation in Cardioembolic stroke patients with AF should be done preferably after 48 hours, taking into consideration the cardioembolic stroke risk recurrence versus HT risk. The treatment included DOAC or VKA anticoagulation (warfarin). They suggest restarting anticoagulation after two days in patients with small strokes, 7-10 days in those with moderate-sized strokes, and 10-14 days in those with large strokes. In the case of multiple infarcts, they recommend considering the size of the largest. In the presence of hemorrhagic transformation, anticoagulation is usually delayed, mainly in large infarcts, for 30 days^29^.

### Resumption of OAC after anticoagulation-related intracerebral hemorrhage

When considering resuming anticoagulation in a patient with hemorrhagic transformation due to OAC we need to analyze the risk of thromboembolism and the risk of enlargement of cerebral hematoma. 

Fiorelli et al. classified the Hemorrhagic Transformation according to the volume of hematoma: HI1, HI2, PH1 and PH2:

- HI 1 Hemorrhage infarction type 1 - Small hyperdense petechiae;
- HI2 - Hemorrhage infarction type 2- More confluent hyperdensity throughout the infarct zone; without mass effect;
- PH 1- Parenchymal hematoma type 1 - Homogeneous hyperdensity occupying <30% of the infarct zone; some mass effect;
- PH 2 - Parenchymal hematoma type 2 - Homogeneous hyperdensity occupying >30% of the infarct zone; significant mass effect.

Kuramatsu et al. performed a retrospective cohort study in Germany and included 1176 patients with anticoagulation-related intracerebral hemorrhage and patients with AF and other cardiomyopathies^30^.

In 853 patients they analyzed hemorrhage enlargement and in 719 oral anticoagulation resumption. Intracerebral hemorrhage enlargement was observed in 307 of 853 patients (36%). Factors that contributed to reducing the risk of hemorrhage enlargement were: rapid reversal of anticoagulation for INR levels <1,3 (within four hours) and rigorous blood pressure control with systolic BP levels <160mmHg within four hours. 

Oral anticoagulation resumption was done in only 172 of 719 patients and was associated with a lower risk of ischemic stroke. OAC resumption occurred on average 31 days after the HT^30^.

Patients with previous cerebral microbleeds or previous lobar hemorrhage suggestive of amyloid angiopathy or without blood pressure control or large ischemic stroke present a major risk of HT or recurrence of cerebral bleeding after the resumption of anticoagulation^31^. In these patients, anticoagulants must be used with caution. 

Mac Grory suggests that patients with mechanical heart valves have a higher potential for cardiac embolization. They suggest, except in cases of large-size cerebral infarction, that these patients should receive early anticoagulation within four to seven days, and the occurrence of cerebral hemorrhage enlargement should be monitored^29^. Another indication was thrombus in the left atrium or left ventricle and malignancy and stroke. 

Mac Grory observed that it is safe to reintroduce anticoagulation in patients with small infarcts and HT after two days. However, in patients with moderate-size cerebral infarction, they recommend delaying anticoagulation for seven to 28 days. In these cases, HT type PH2 was observed even 28 days after the stroke. In cases of large cerebral infarction, they suggest delaying anticoagulation for 10 to 42 days for the same reason. 

According to Lansberg et al., annual hemorrhagic transformation in patients using oral anticoagulation varies between 0.6% to 1%^32^.

In a Danish study, 1725 patients with acute ischemic stroke anticoagulation resumption was done between two to 10 weeks later.

Researchers recommend delaying ACO resumption after an ICH for at least two weeks, usually restarting after around four weeks. However, in patients with cerebellar ICH it would be better to wait eight to 10 weeks and in patients with mechanical prosthetic valves and small ICH ACO should be reintroduced after two weeks^33^ ^,^ ^34^.

### Embolic stroke of undetermined source (ESUS)

For embolic stroke of undetermined source (ESUS) patients, two trials, NAVIGATE - ESUS (rivaroxaban and aspirin) and RES-PECT ESUS, compared the effect of anticoagulation and aspirin^36^ ^-^ ^38^.

In the NAVIGATE- ESUS trial, rivaroxaban was no better than aspirin with regard to the prevention of recurrent stroke in ESUS patients and was associated with a higher risk of bleeding^37^.

### Comparison of LMWH/Heparin Bridging Versus No Bridging Therapy to oral anticoagulation

According to Yaghi et al patients treated with LMWH/heparin bridging presented a significantly higher rate of symptomatic cerebral hemorrhage (4.4% versus 1.0%)^35^.

Bridging could be considered in patients with small infarcts and intracardiac thrombus.

### Stroke and malignancy

Stroke and malignancy can occur simultaneously, while thrombosis is a well-known complication in cancer.

Although the number of publications on cancer-related stroke has recently increased, there are no evidence-based guidelines for treatment of cancer-related stroke. The effects of DOACs were reported in only a few cases, most of all retrospective studies, but the efficacy and safety of DOACs in cancer-related stroke have not yet been settled by comprehensive studies.

A large retrospective study demonstrated the efficacy and safety of DOACs as compared with warfarin in patients with atrial fibrillation and active cancer. DOACs were more effective in preventing venous thromboembolism (VTE) than warfarin. They were similar or superior to warfarin in stroke prevention and less major bleeding, though lacked statistical significance^39^.

A clinical trial, ENGAGE AF-TIMI 48, studied 1153 atrial fibrillation patients with new or recurrent cancer treated with warfarin or edoxaban. The results show that high-dose and low-dose edoxaban decreased stroke and systemic embolic events as compared with warfarin, but there was no statistical significance and a low tendency to major bleeding^40^.

A recent meta-analysis comparing available data regarding the efficacy and safety of DOACs versus warfarin in cancer patients with nonvalvular AF. In comparison to VKA, DOACs were associated with a significant reduction in the rates of thromboembolic events and major bleeding complications in patients with AF and cancer^41^.

The occurrence of venous thromboembolism (VTE) as a stroke complication is extremely frequent in both. Anticoagulant therapy is the cornerstone of treatment. Acute treatment for patients with no severe renal insufficiency or contraindication to anticoagulation is the use of low molecular weight heparin; to avoid using injections, apixaban is an alternative, based upon a single randomized trial with similar efficacy and safety compared with LMW heparin^42^.

### Antiphospholipid antibodies syndrome

Antiphospholipid syndrome is an autoimmune disease that is associated with a high rate of recurrent thrombosis such as stroke, pulmonary thromboembolism, peripheral venous thrombosis and sometimes miscarriages.

According Sapporo criteria to fulfill the diagnosis of APS a patient must present one clinical criterion and one laboratorial criterion (the presence of at least one of antiphospholipid antibodies: lupus anticoagulant, anticardiolipin antibodies and anti beta2 glycoprotein I antibodies). A challenging situation is a life-threatening multiorgan thrombosis, Catastrophic APS. 

EULAR recommends anticoagulation after a first arterial thrombosis with VKA drugs (warfarin) with a target INR between 2 and 3. DOACs are not recommended mainly in patients with triple APL positive (three APL antibodies positive) and arterial events. 

Patients with catastrophic APS are treated with anticoagulation with heparin associated with glucocorticoids and plasma exchange or intravenous immunoglobulin^43^.

In conclusion, anticoagulation in stroke treatment remains a challenge. It is important always to keep in mind anticoagulation and analyze the patient’s risk of stroke recurrence and HT. However, even in patients with a high risk of HT it is important not to exclude this option, but to consider delaying it, mainly in patients with high CHA_2_DS_2_VASc.

## References

[B1] Ministério da Saúde Mortalidade em doenças cerebrovasculares.

[B2] Silva GS, Rocha ECA, Pontes-Neto OM, Martins SO (2018). Stroke care services in Brazil. J Stroke Med.

[B3] Fuster V (1999). Epidemic of cardiovascular disease and stroke: the three main challenges. Presented at the 71st scientific sessions of the American Heart Association. Dallas, Texas. Circulation.

[B4] Benjamin EJ, Blaha MJ, Chiuve SE, Cushman M, Das SR, Deo R (2017). Heart disease and stroke statistics-2017 update: a report from the American Heart Association. Circulation.

[B5] Jadhav AP, Molyneaux BJ, Hill MD, Jovin TG (2018). Care of the post-thrombectomy patient. Stroke.

[B6] Powers WJ, Rabinstein AA, Ackerson T, Adeoye OM, Bambakidis NC, Becker K (2019). Guidelines for the Early Management of Patients with Acute Ischemic Stroke: 2019 Update to the 2018 Guidelines for the Early Management of Acute Ischemic Stroke: A Guideline for Healthcare Professionals from the American Heart Association/American Stroke Association. Stroke.

[B7] Low molecular weight heparinoid (1998). ORG 10172 (danaparoid), and outcome after acute ischemic stroke: a randomized controlled trial The Publications Committee for the Trial of ORG 10172 in Acute Stroke Treatment (TOAST) Investigators. JAMA.

[B8] Wang QS, Chen C, Chen XY, Han JH, Soo Y, Leung TW (2012). Low-molecular-weight heparin versus aspirin for acute ischemic stroke with large artery occlusive disease: subgroup analyses from the Fraxiparin in Stroke Study for the treatment of ischemic stroke (FISS-tris) study. Stroke.

[B9] The International Stroke Trial (1997). (IST): A randomised trial of aspirin, subcutaneous heparin, both, or neither among 19435 patients with acute ischaemic stroke. International Stroke Trial Collaborative Group. Lancet.

[B10] Paciaroni M, Agnelli G, Micheli S, Caso V (2007). Efficacy and safety of anticoagulant treatment in acute cardioembolic stroke: a meta-analysis of randomized controlled trials. Stroke.

[B11] Mokin M, Kass-Hout T, Kass-Hout O, Radovic V, Siddiqui AH, Levy EI (2013). Intravenous heparin for the treatment of intraluminal thrombus in patients with acute ischemic stroke: a case series. J Neurointerv Surg.

[B12] Vellimana AK, Kadkhodayan Y, Rich KM, Cross DT, Moran CJ, Zazulia AR (2013). Symptomatic patients with intraluminal carotid artery thrombus: outcome with a strategy of initial anticoagulation. J Neurosurg.

[B13] Engelter ST, Brandt T, Debette S, Caso V, Lichy C, Pezzini A (2007). Antiplatelets versus anticoagulation in cervical artery dissection. Stroke.

[B14] Ma A, Kase CS, Shoamanesh A, Abdalkader M, Pikula A, Sathya A (2021). Stroke and thromboprophylaxis in the Era of COVID-19. J Stroke Cerebrovasc Dis.

[B15] Chiasakul T, De Jesus E, Tong J, Chen Y, Crowther M, Garcia D (2019). Inherited thrombophilia and the risk of arterial ischemic stroke: a systematic review and meta‐analysis. J Am Heart Assoc.

[B16] Kamel H, Healey JS (2017). Cardioembolic stroke. Circ Res.

[B17] Aguilar MI, Hart R, Pearce LA (2007). Oral anticoagulants versus antiplatelet therapy for preventing stroke in patients with non-valvular atrial fibrillation and no history of stroke or transient ischemic attacks. Cochrane Database Syst Rev.

[B18] Avezum A, Lopes RD, Schulte PJ, Lanas F, Gersh BJ, Hanna M (2015). Apixaban in comparison with warfarin in patients with atrial fibrillation and valvular heart disease: findings from the apixaban for reduction in stroke and other thromboembolic events in atrial fibrillation (ARISTOTLE) trial. Circulation.

[B19] Connolly SJ, Ezekowitz MD, Yusuf S, Eikelboom J, Oldgren J, Parekh A (2009). Dabigatran versus warfarin in patients with atrial fibrillation. N Engl J Med.

[B20] Halperin JL, Hankey GJ, Wojdyla DM, Piccini JP, Lokhnygina Y, Patel MR (2014). Efficacy and safety of rivaroxaban compared with warfarin among elderly patients with nonvalvular atrial fibrillation in the Rivaroxaban Once Daily, Oral, Direct Factor Xa Inhibition Compared with Vitamin K Antagonism for Prevention of Stroke and Embolism Trial in Atrial Fibrillation (ROCKET AF). Circulation.

[B21] Kato ET, Giugliano RP, Ruff CT, Koretsune Y, Yamashita T, Kiss RG (2016). Efficacy and Safety of Edoxaban in Elderly Patients with Atrial Fibrillation in the ENGAGE AF-TIMI 48 Trial. J Am Heart Assoc.

[B22] January CT, Wann LS, Alpert JS, Calkins H, Cigarroa JE, Cleveland JC (2014). 2014 AHA/ACC/HRS guideline for the management of patients with atrial fibrillation: A report of the American College of Cardiology/American Heart Association Task Force on Practice Guidelines and the Heart Rhythm Society. J Am Coll Cardiol.

[B23] Lip GY, Lane DA (2015). Stroke prevention in atrial fibrillation: a systematic review. JAMA.

[B24] Kirchhof P, Benussi S, Kotecha D, Ahlsson A, Atar D, Casadei B (2016). 2016 ESC Guidelines for the management of atrial fibrillation developed in collaboration with EACTS. Eur Heart J.

[B25] Hart RG, Coull BM, Hart D (1983). Early recurrent embolism associated with nonvalvular atrial fibrillation: a retrospective study. Stroke.

[B26] Pisters R, Lane DA, Nieuwlaat R, de Vos CB, Crijns HJ, Lip GY (2010). A novel user-friendly score (HAS-BLED) to assess 1-year risk of major bleeding in patients with atrial fibrillation: The Euro Heart Survey. Chest.

[B27] Paciaroni M, Agnelli G, Falocci N, Caso V, Becattini C, Marcheselli S (2015). Early recurrence and cerebral bleeding in patients with acute ischemic stroke and atrial fibrillation: Effect of anticoagulation and its timing: The RAF Study. Stroke.

[B28] Hornig CR, Bauer T, Simon C, Trittmacher S, Dorndorf W (1993). Hemorrhagic transformation in cardioembolic cerebral infarction. Stroke.

[B29] Mac Grory B, Flood S, Schrag M, Paciaroni M, Yaghi S (2019). Anticoagulation resumption after stroke from atrial fibrillation. Curr Atheroscler Rep.

[B30] Fiorelli M, Bastianello S, von Kummer R, del Zoppo GJ, Larrue V, Lesaffre E (1999). Hemorrhagic transformation within 36 hours of a cerebral infarct: relationships with early clinical deterioration and 3-month outcome in the European Cooperative Acute Stroke Study I (ECASS I) cohort. Stroke.

[B31] Kuramatsu JB, Gerner ST, Schellinger PD, Glahn J, Endres M, Sobesky J (2015). Anticoagulant reversal, blood pressure levels, and anticoagulant resumption in patients with anticoagulation-related intracerebral hemorrhage. JAMA.

[B32] Lansberg MG, O'Donnell MJ, Khatri P, Lang ES, Nguyen-Huynh MN, Schwartz NE (2012). Antithrombotic and thrombolytic therapy for ischemic stroke: Antithrombotic Therapy and Prevention of Thrombosis, 9th ed: American College of Chest Physicians Evidence-Based Clinical Practice Guidelines. Chest.

[B33] Becattini C, Sembolini A, Paciaroni M (2016). Resuming anticoagulant therapy after intracerebral bleeding. Vascul Pharmacol.

[B34] Nielsen PB, Larsen TB, Skjøth F, Gorst-Rasmussen A, Rasmussen LH, Lip GYH (2015). Restarting anticoagulant treatment after intracranial hemorrhage in patients with atrial fibrillation and the impact on recurrent stroke, mortality, and bleeding: a nationwide cohort study. Circulation.

[B35] Yaghi S, Mistry E, Liberman AL, Giles J, Asad SD, Liu A (2020). Antigoagulant type and early recurrence in cardioembolic stroke. Stroke.

[B36] Hart RG, Catanese L, Perera KS, Ntaios G, Connolly SJ (2017). Embolic stroke of undetermined source: a systematic review and clinical update. Stroke.

[B37] Hart RG, Sharma M, Mundl H, Kasner SE, Bangdiwala SI, Berkowitz SD (2018). Rivaroxaban for stroke prevention after embolic stroke of undetermined source. N Engl J Med.

[B38] Diener HC, Sacco RL, Easton JD, Granger CB, Bernstein RA, Uchiyama S (2019). Dabigatran for prevention of stroke after embolic stroke of undetermined source. N Engl J Med.

[B39] Shah S, Norby FL, Datta YH, Lutsey PL, MacLehose RF, Chen LY (2018). Comparative effectiveness of direct oral anticoagulants and warfarin in patients with cancer and atrial fibrillation. Blood Adv.

[B40] Fanola CL, Ruff CT, Murphy SA, Jin J, Duggal A, Babilonia NA (2018). Efficacy and Safety of Edoxaban in Patients with Active Malignancy and Atrial Fibrillation: Analysis of the ENGAGE AF - TIMI 48 Trial. J Am Heart Assoc.

[B41] Mariani MV, Magnocavallo M, Straito M, Piro A, Severino P, Iannucci G (2021). Direct oral anticoagulants versus vitamin K antagonists in patients with atrial fibrillation and cancer a meta-analysis. J Thromb Thrombolysis.

[B42] Agnelli G, Becattini C, Meyer G, Muñoz A, Huisman MV, Connors JM (2020). Apixaban for the Treatment of Venous Thromboembolism Associated with Cancer. N Engl J Med.

[B43] Tektonidou MG, Andreoli L, Limper M, Amoura Z, Cervera R, Costedoat-Chalumeau N (2019). EULAR recommendations for the management of antiphospholipid syndrome in adults. Ann Rheum Dis.

